# Supplementary material for: Genome-Wide Identification and Characterization of RNA/DNA Differences Associated with Fusarium graminearum Infection in Wheat
Source: Int J Mol Sci. 2022 Jul 20;23(14):7982. doi: 10.3390/ijms23147982 (PMC9316857; doi:10.3390/ijms23147982)
Supplement: Supplementary file 1 [file ijms-23-07982-s001.zip › Supplementary Figures.pdf]

Supplementary Figures

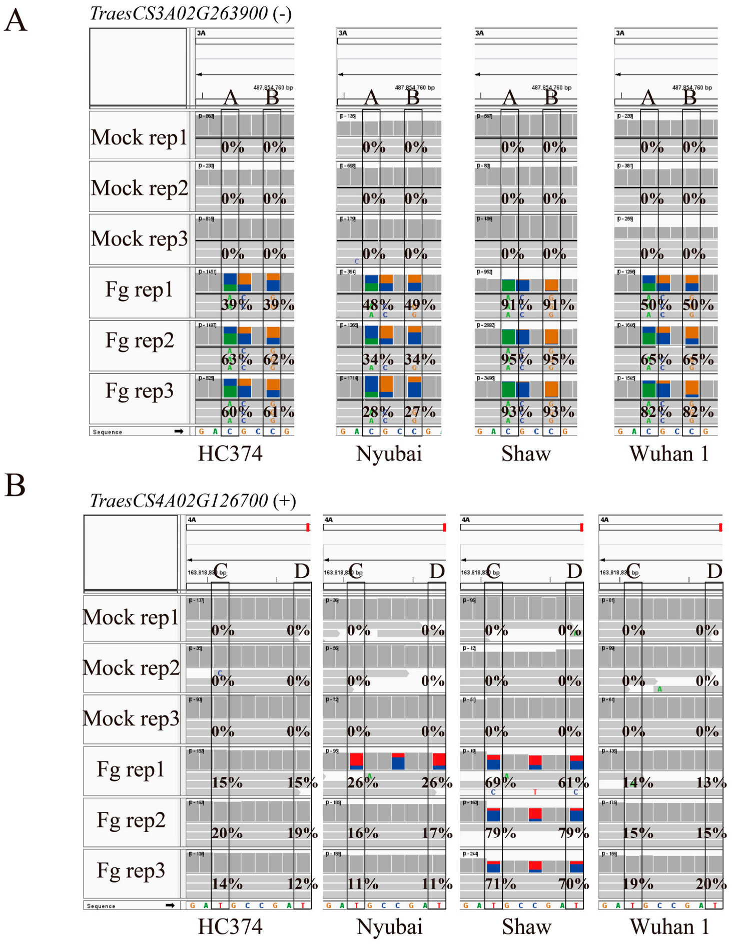

Figure S1 IGV results of RDD events in TraesCS3A02G263900 (A) and TraesCS4A02G126700 (B). RDD of A-D indicate chr3A_487854757, chr3A_487854760, chr4A_163818831 and chr4A_163818837, respectively. The numbers on reads represent the editing efficiency of RDD sites in each sample. Mock: Control group. Fg: Treatment group.

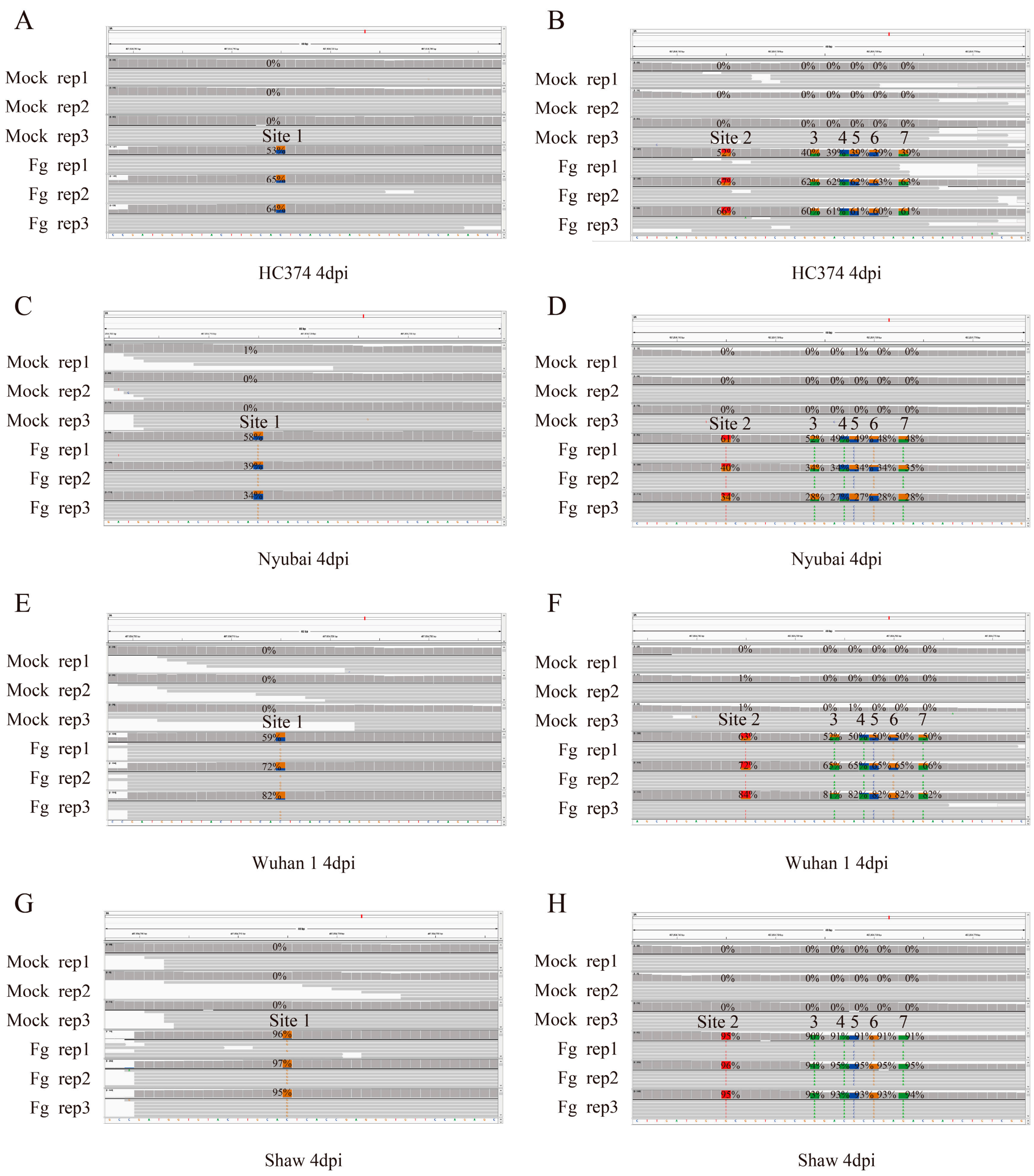

Figure S2. IGV results of RDD events in TraesCS3A02G263900. (**A**) Site 1 in 4 dpi of HC374. (**B**) Site 2-7 in 4 dpi of HC374. (**C**) Site 1 in 4 dpi of Nyubai. (**D**) Site 2-7 in 4 dpi of Nyubai. (**E**) Site 1 in 4 dpi of Wuhan 1. (**F**) Site 2-7 in 4 dpi of Wuhan 1. (**G**) Site 1 in 4 dpi of Shaw. (**H**) Site 2-7 in 4 dpi of Shaw. The numbers on reads represent the editing efficiency of RDD sites in each sample. Mock: Control group. Fg: Treatment group.

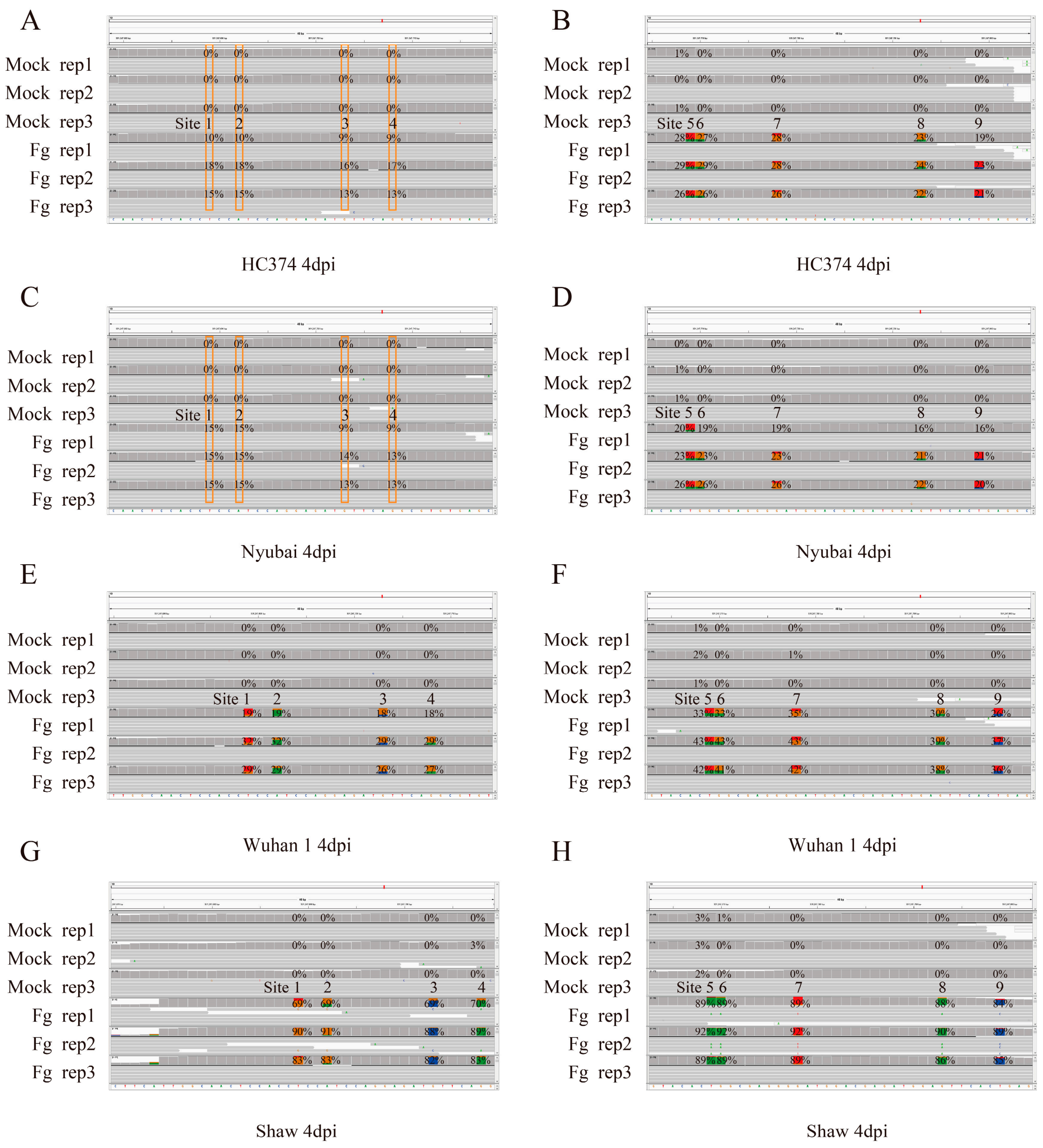

Figure S3. IGV results of RDD events in TraesCS1D02G258800. (**A**) Site 1-4 in 4 dpi of HC374. (**B**) Site 5-9 in 4 dpi of HC374. (**C**) Site 1-4 in 4 dpi of Nyubai. (**D**) Site 5-9 in 4 dpi of Nyubai. (**E**) Site 1-4 in 4 dpi of Wuhan 1. (**F**) Site 5-9 in 4 dpi of Wuhan 1. (**G**) Site 1-4 in 4 dpi of Shaw. (**H**) Site 5-9 in 4 dpi of Shaw. The numbers on reads represent the editing efficiency of RDD sites in each sample. Mock: Control group. Fg: Treatment group.

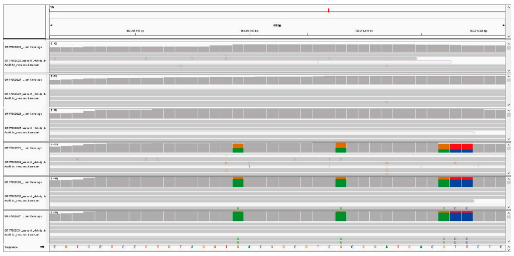

chr1A_362209999,chr1A_362210008,
chr1A_362210017,chr1A_362210018,chr1A_362210019

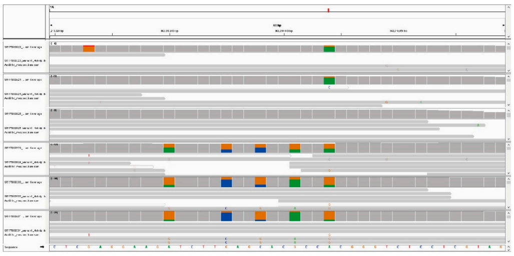

chr1A_362210030,chr1A_362210035,
chr1A_362210038,chr1A_362210041,chr1A_362210044

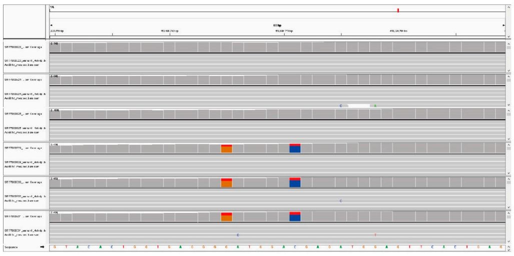

chr1A_452020765,chr1A_452020771

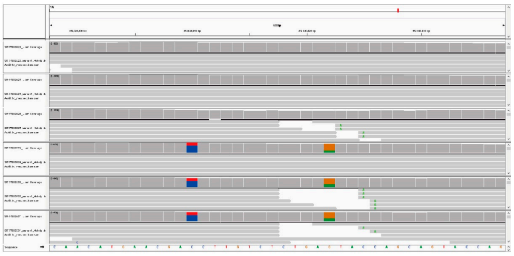

chr1A_452020810,chr1A_452020822

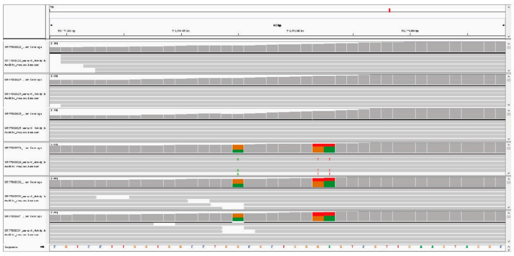

chr1B_512174375,chr1B_512174382,chr1B_512174383

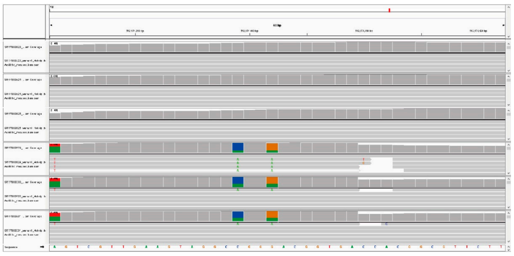

chr1B_512174399,chr1B_512174402

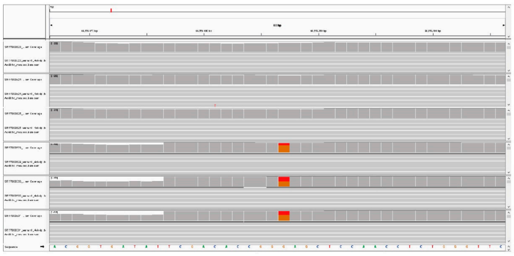

chr1D_66556087

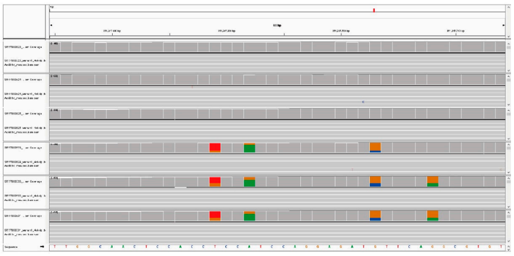

chr1D_351247689,chr1D_351247692,chr1D_351247703,
chr1D_351247708

Figure S4. IGV results of 24 RDD sites of TraesCS1A02G201300, TraesCS1A02G258800, TraesCS1B02G294300, TraesCS1D02G083600 and TraesCS1D02G258800.

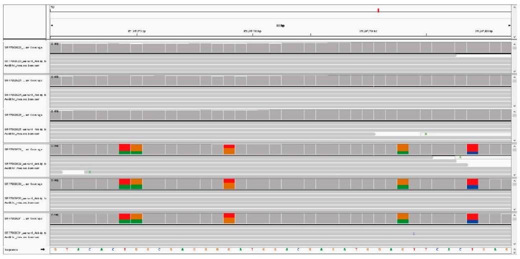

chr1D_351247769,chr1D_351247770,chr1D_351247778,
chr1D_351247793,chr1D_351247799

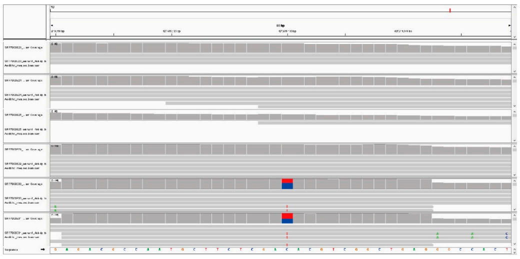

chr1D_427619130

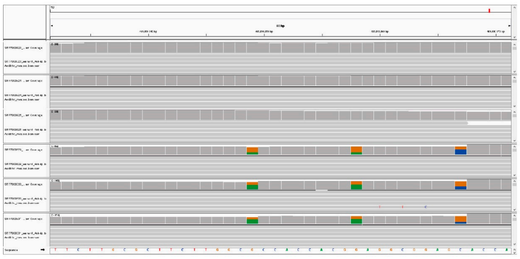

chr1D_469890349,chr1D_469890358,chr1D_469890367

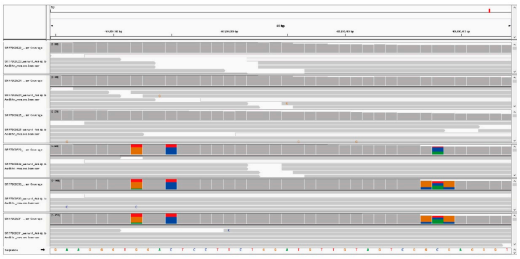

chr1D_469890382,chr1D_469890385,chr1D_469890408

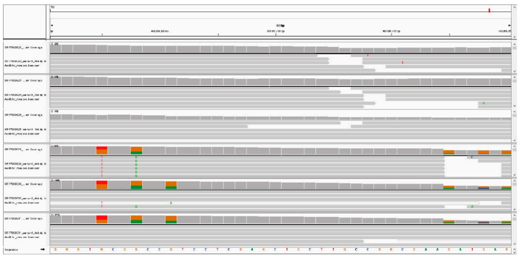

chr1D_469890415,chr1D_469890418

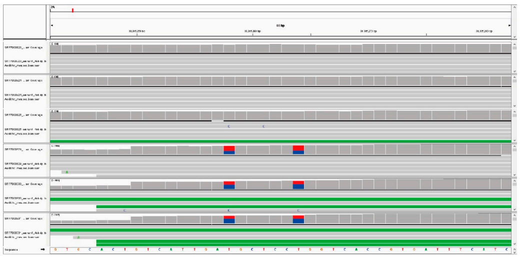

chr2A_38305258,chr2A_38305264

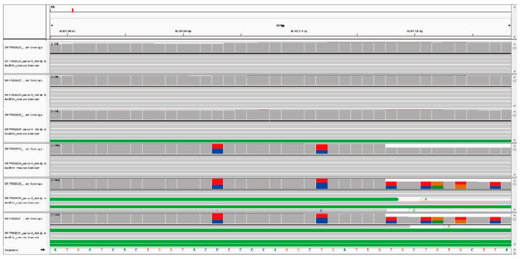

chr2A_38305303,chr2A_38305312

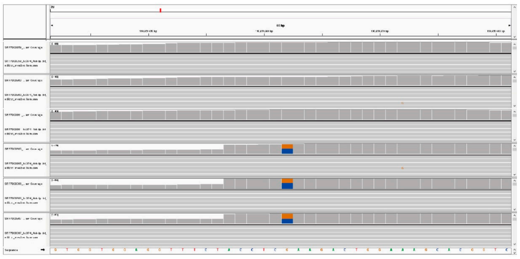

chr2B_190659662

Figure S5. IGV results of 19 RDD sites of TraesCS1D02G258800, TraesCS1D02G338400, TraesCS1D02G405400, TraesCS2A02G083300 and TraesCS2B02G209100.

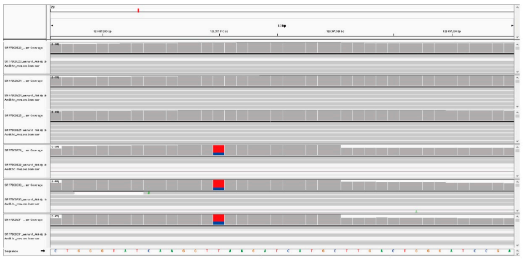

chr2D_123097910

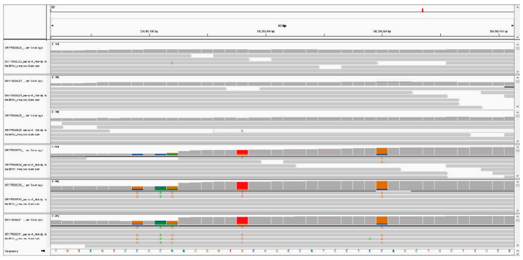

chr2D_520582908

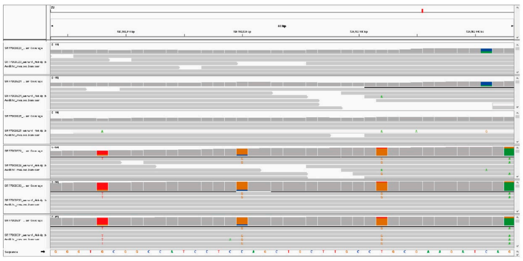

chr2D_520582920

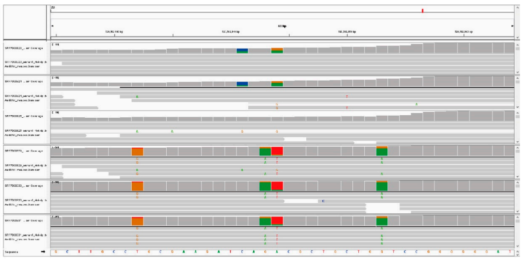

chr2D_520582943

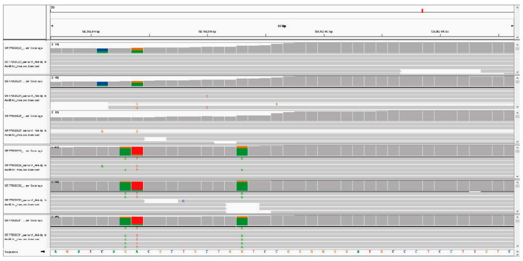

chr2D_520582953

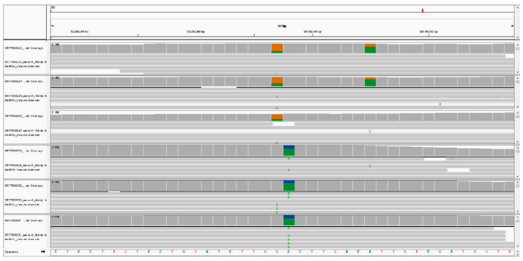

chr2D_520582988

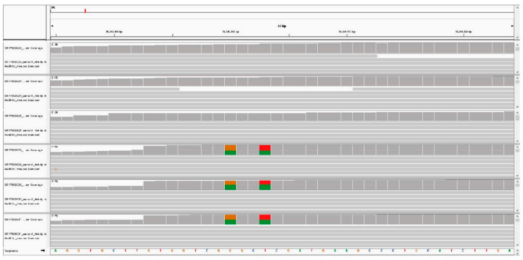

chr3A_56390500,chr3A_56390503

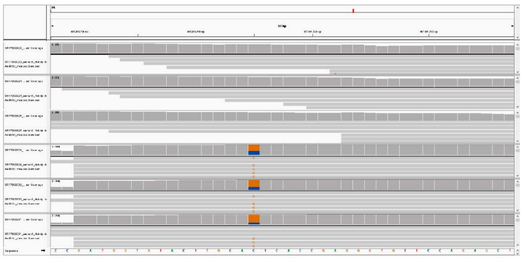

chr3A_487854715

Figure S6. IGV results of 9 RDD sites of TraesCS2D02G179300, TraesCS2D02G405500, TraesCS3A02G087300 and TraesCS3A02G263900.

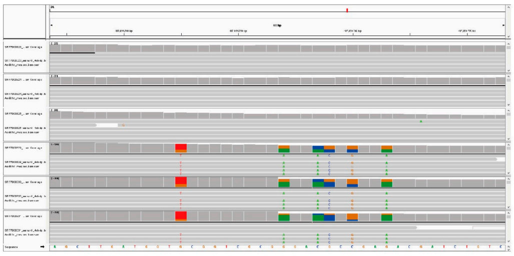

chr3A_487854745,chr3A_487854754,chr3A_487854757,
chr3A_487854758,chr3A_487854760,chr3A_487854763

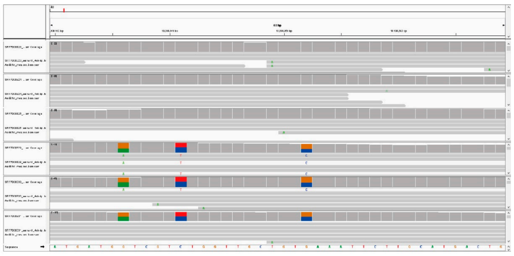

chr3D_18908936,chr3D_18908941,chr3D_18908952

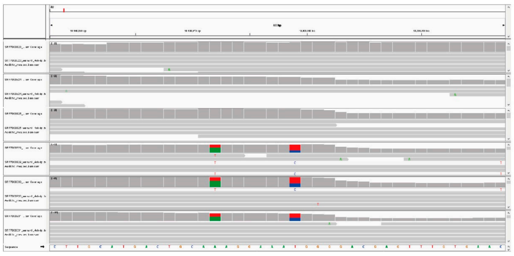

chr3D_18908972,chr3D_18908979

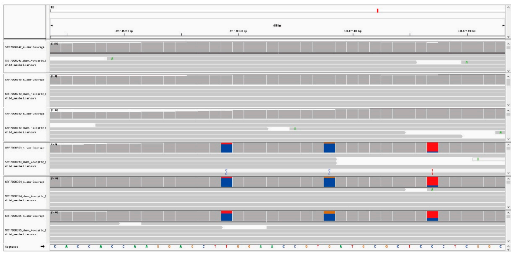

chr3D_441147419,chr3D_441147428

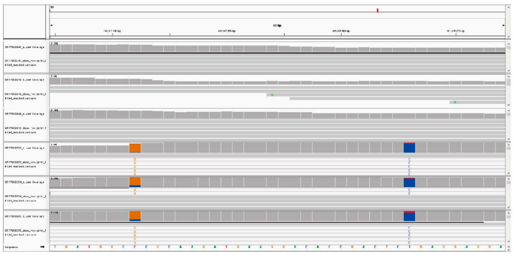

chr3D_441147542,chr3D_441147566

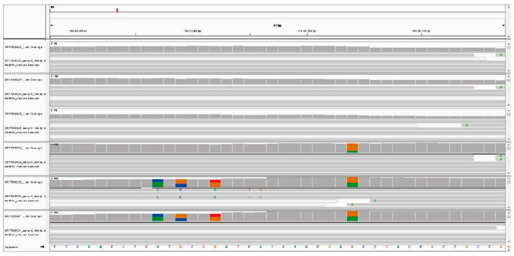

chr4A_109424377,chr4A_109424379,
chr4A_109424382,chr4A_109424394

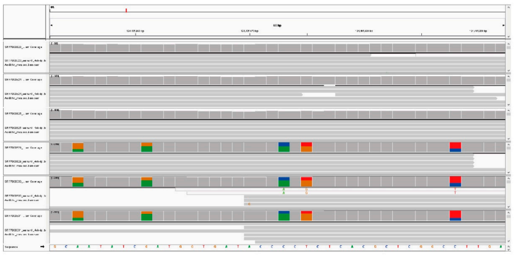

chr4A_124405655,chr4A_124405661,
chr4A_124405673,chr4A_124405675,chr4A_124405688

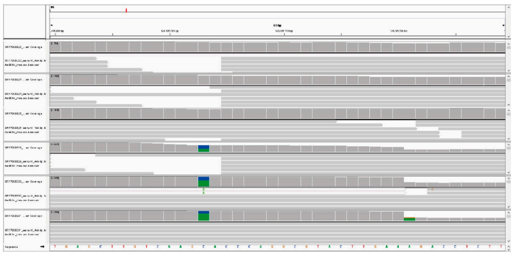

chr4A_124405703

Figure S7. IGV results of 25 RDD sites of TraesCS3A02G263900, TraesCS3D02G049300, TraesCS3D02G328300, TraesCS4A02G097900 and TraesCS4A02G107600.

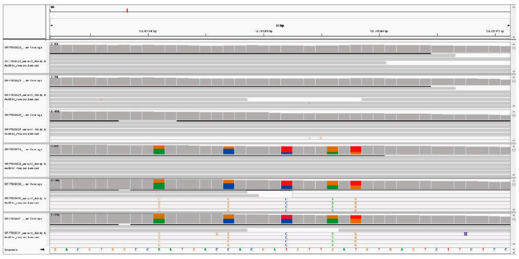

chr4A_124405841,chr4A_124405847,chr4A_124405852,
chr4A_124405856,chr4A_124405858

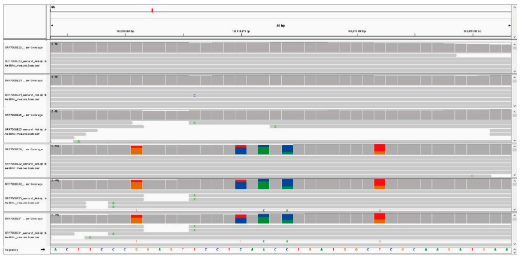

chr4A_163818861,chr4A_163818870,chr4A_163818872,
chr4A_163818874,chr4A_163818882

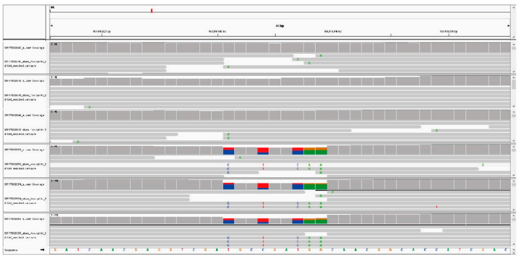

chr4A_163818831,chr4A_163818834,chr4A_163818837,
chr4A_163818838,chr4A_163818839

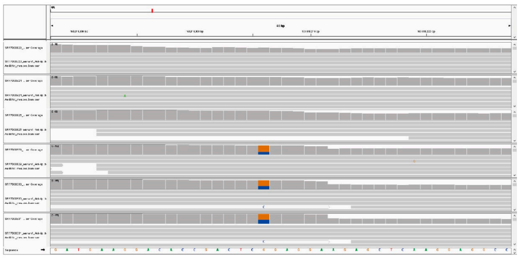

chr4A_163818906

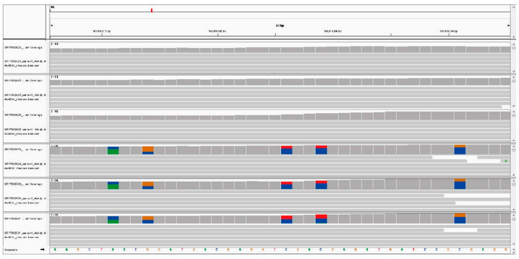

chr4A_163819011,chr4A_163819014,chr4A_163819026,
chr4A_163819029,chr4A_163819041

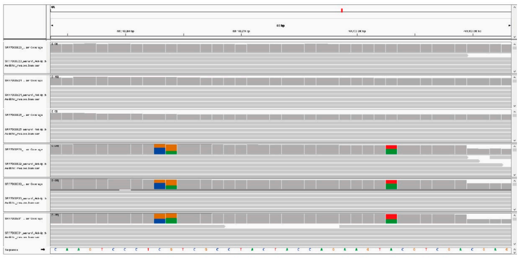

chr4A_469102263,chr4A_469102264,chr4A_469102283

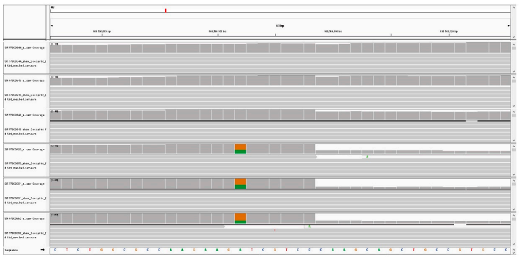

chr4B_168566902

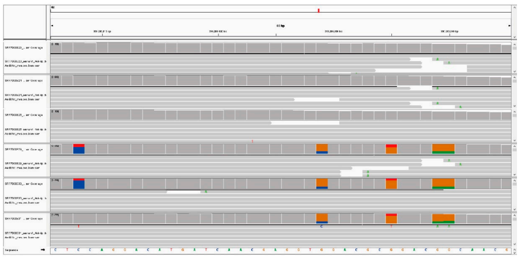

chr4B_390280808,chr4B_390280829,chr4B_390280835,
chr4B_390280839,chr4B_390280840

Figure S8. IGV results of 30 RDD sites of TraesCS4A02G107600, TraesCS4A02G126700, TraesCS4A02G190500, TraesCS4B02G128700 and TraesCS4B02G178200.

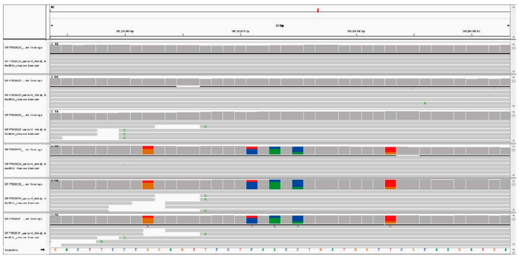

chr4B_390280862,chr4B_390280871,chr4B_390280873,
chr4B_390280875,chr4B_390280883

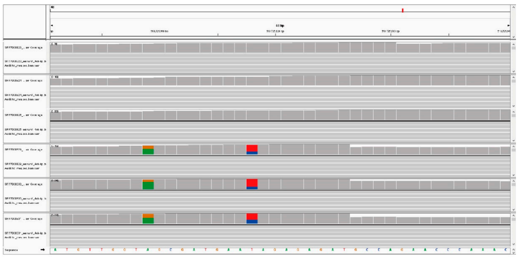

chr4B_513725918

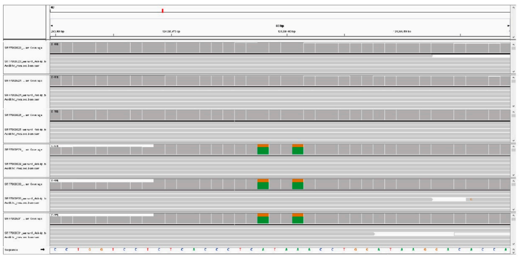

chr4D_124260478,chr4D_124260481

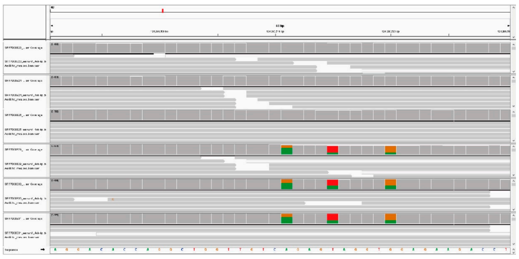

chr4D_124260511,chr4D_124260515,chr4D_124260520

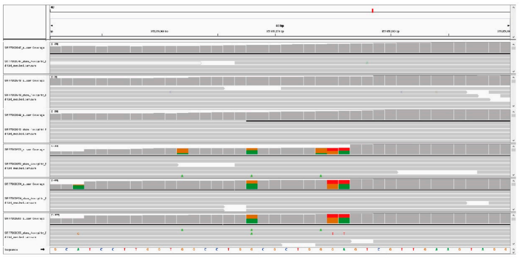

chr4D_3558599680

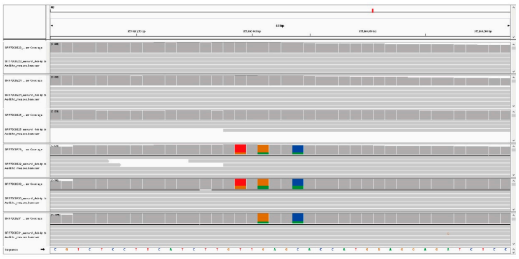

chr4D_355860059,chr4D_355860061,chr4D_355860064

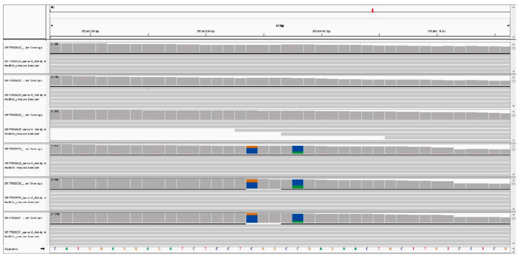

chr4D_355860084,chr4D_355860088

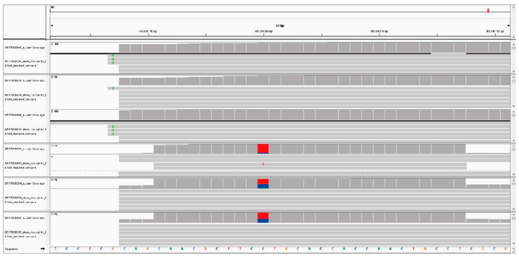

chr4D_483138160

Figure S9. IGV results of 18 RDD sites of TraesCS4B02G178200, TraesCS4B02G248500, TraesCS4D02G140800, TraesCS4D02G207500 and TraesCS4D02G319400.

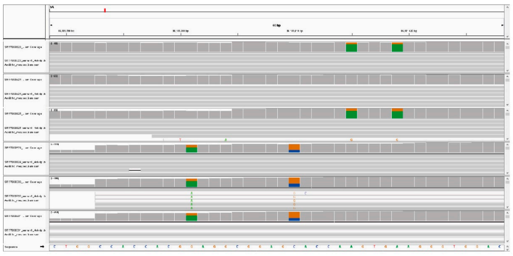

chr5A_86181601,chr5A_86181610

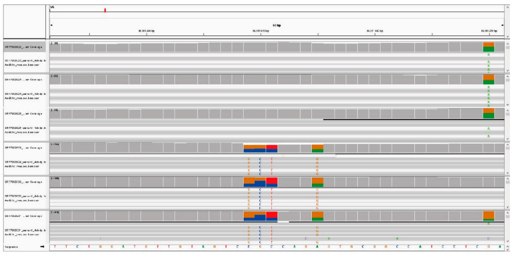

chr5A_86181649,chr5A_86181650,chr5A_86181651, chr5A_86181655

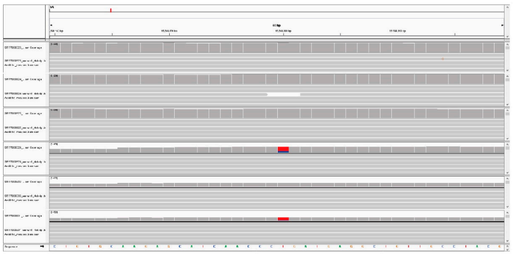

chr5A_95568180

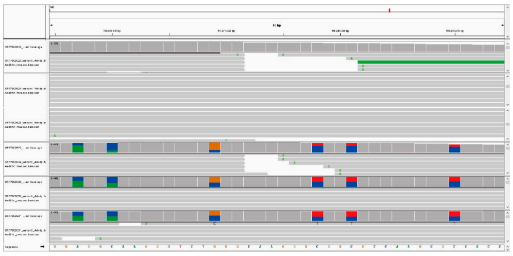

chr5B_530613607,chr5B_530613610,chr5B_530613619, chr5B_530613628,chr5B_530613631,chr5B_530613640

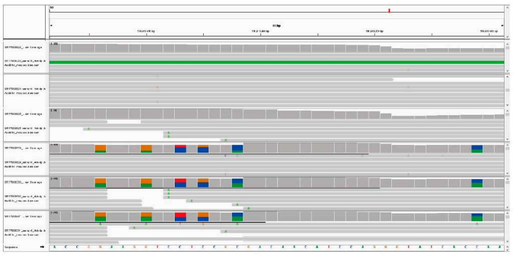

chr5B_530613646,chr5B_530613650,chr5B_530613653, chr5B_530613655,chr5B_530613658,chr5B_530613679

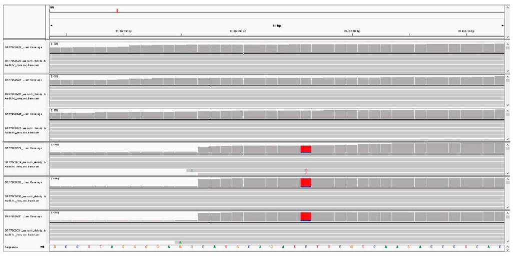

chr6A_91424406

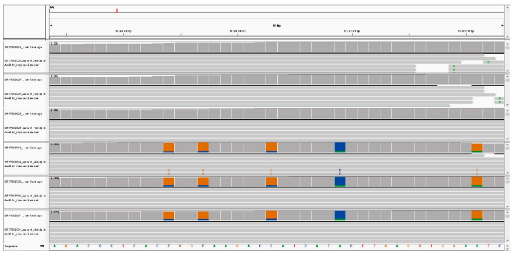

chr6A_91424424,chr6A_91424427,chr6A_91424433, chr6A_91424439,chr6A_91424451

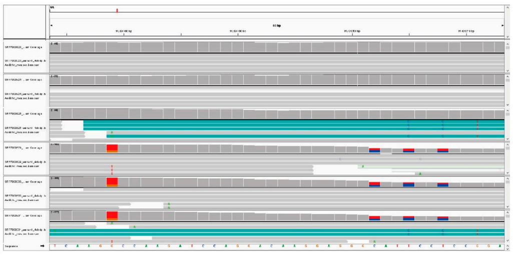

chr6A_91424479,chr6A_91424508

Figure S10. IGV results of 27 RDD sites of TraesCS5A02G073800, TraesCS5A02G078000, TraesCS5B02G349100, TraesCS6A02G119700 and TraesCS6A02G119800.

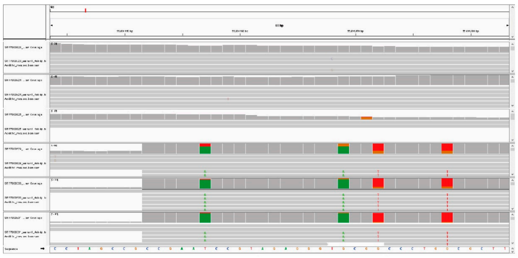

chr6B_55694957,chr6B_55694969,chr6B_55694978

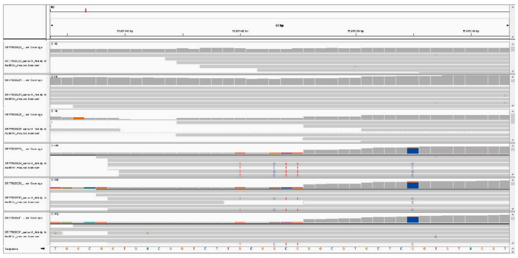

chr6B_55695035

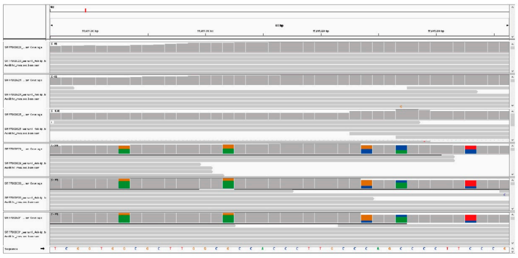

chr6B_55695203,chr6B_55695212,chr6B_55695224,
chr6B_55695227,chr6B_55695233

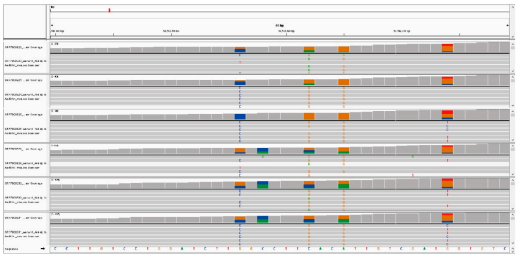

chr6B_92532418

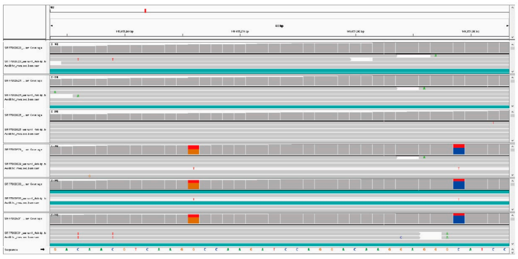

chr6B_149055266,chr6B_149055289

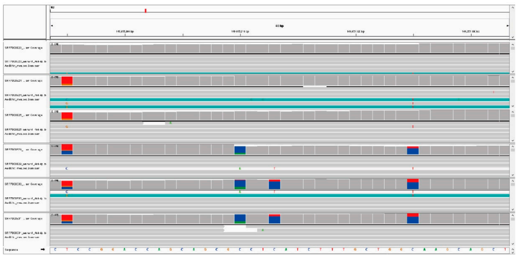

chr6B_149055310

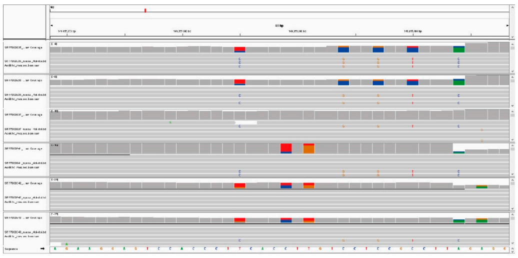

chr6B_149055389,chr6B_149055391

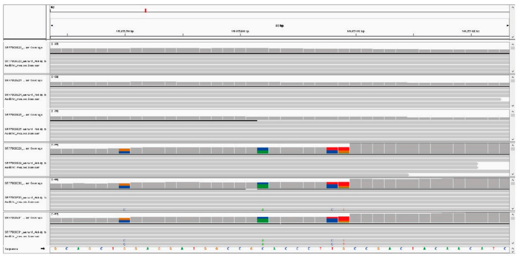

chr6B_149055802

Figure S11. IGV results of 16 RDD sites of TraesCS6B02G079200, TraesCS6B02G110300 and TraesCS6B02G148000.

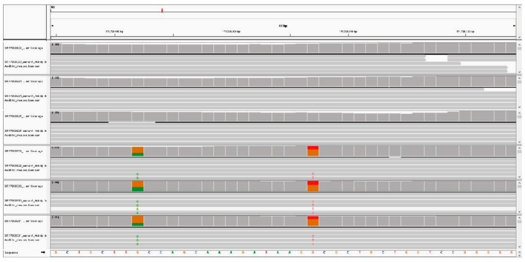

chr6B_171728092,chr6B_171728107

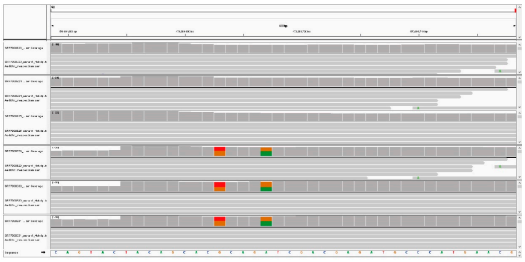

chr6D_470684693,chr6D_470684697

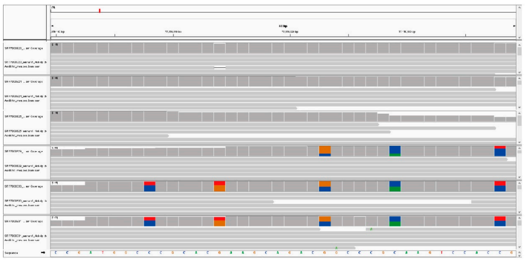

chr7A_77150114,chr7A_77150123,chr7A_77150129,
chr7A_77150138

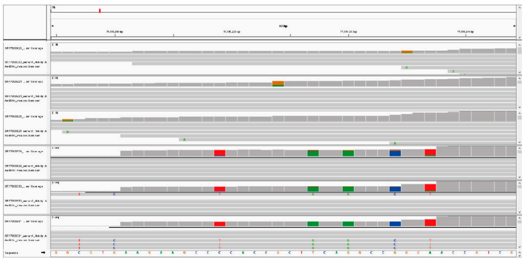

chr7A_77150219,chr7A_77150227,chr7A_77150237

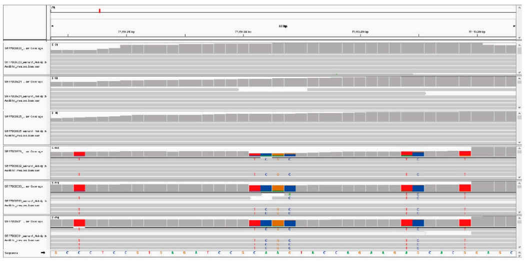

chr7A_77150263

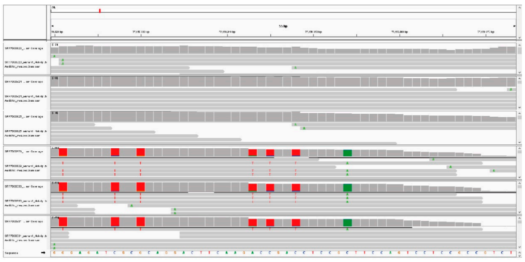

chr7A_77150321,chr7A_77150327,chr7A_77150330,
chr7A_77150343,chr7A_77150345,chr7A_77150348

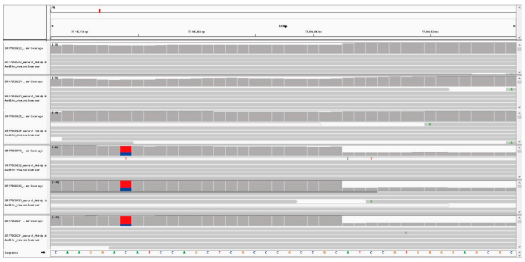

chr7A_77150474

Figure S12. IGV results of 19 RDD sites of TraesCS6B02G164600, TraesCS6D02G401900 and TraesCS7A02G119700.
